# Production, characterization, and antimicrobial activity of polyhydroxyalkanoates synthesized by *Bacillus* species against skin pathogens

**DOI:** 10.1039/d5ra04375a

**Published:** 2025-09-24

**Authors:** Manal, Faiqa Munir, Waseem Safdar, Muhammad Abu bakr shabbir, Saeed Ahmed, Muhammad Tariq Navid, Mahwish Ali, Iftikhar Ahmed

**Affiliations:** a Department of Biological Sciences, National University of Medical Sciences Rawalpindi Pakistan tariq.navid@numspak.edu.pk waseem.safdar@numspak.edu.pk saeed.ahmed@numspak.edu.pk +92 51-9270677 +92 333 6488 302; b Department of Microbiology, University of Veterinary and Animal Sciences Lahore Pakistan; c National Culture Collection of Pakistan (NCCP), Land Resources Research Institute (LRRI), National Agricultural Research Centre (NARC) Park Road Islamabad Pakistan

## Abstract

Polyhydroxyalkanoates (PHAs) are biodegradable polyesters with promising biomedical applications, particularly for combating antibiotic-resistant skin infections, in the development of wound dressings and other healthcare materials. The growing challenge of antibiotic-resistant skin infections has been managed by exploiting PHAs as an alternative to synthetic materials due to their sustainability, eco-friendly nature and antimicrobial functionality. This research used an orthogonal experimental design for PHB optimization, and the results demonstrated that ammonium sulphate (2 g L^−1^), glucose, 10% NaCl, and 67 h incubation maximized PHB yield (46.7%). Citric acid supplementation (2–4 g L^−1^) enhanced acetyl-CoA flux, increasing PHB synthesis. The extracted PHB was characterized using FTIR, ^1^H-NMR, and ^13^C-NMR spectroscopies, confirming the presence of poly-3-hydroxybutyrate (PHB). Thermal and structural characterization of the synthesized PHB confirmed its semi-crystalline nature and enhanced thermal stability. TGA, DSC, and XRD analyses revealed high degradation temperatures, distinct melting and crystallization transitions, and well-defined diffraction peaks, indicating a stable and structurally ordered polymer. PHB exhibited potent antimicrobial activity against skin pathogens, with zones of inhibition of 16 mm for *Staphylococcus aureus*, 14 mm for *S. epidermidis*, and 10 mm for *Candida albicans* at 1000 μg per disk. The MIC values were 400 μg mL^−1^ for *S. aureus*, 600 μg mL^−1^ for *S. epidermidis*, and 1000 μg mL^−1^ for *C. albicans*. Time-kill assays showed complete eradication of *S. aureus* within 8 h. PHB also disrupted biofilms, achieving 60% inhibition (co-incubation) and 70% eradication (preformed biofilms) at MIC concentrations. The XTT assay revealed PHA's dose-dependent *anti*-biofilm activity. Co-incubation with 600 μg mL^−1^ PHB strongly inhibited biofilms (24–55% viability), while 1000 μg mL^−1^ nearly eradicated bacterial biofilms (12–16% viability). PHB also disrupted the preformed biofilms, showing stronger activity against Gram-positive bacteria than against *C. albicans*. This study underscores the potential of *B. megaterium*-derived PHB as an eco-friendly antimicrobial agent against resistant skin pathogens, aligning with global efforts to replace synthetic plastics and antibiotics.

## Abstract 2

Polyhydroxyalkanoates (PHAs) are biodegradable polyesters with promising biomedical applications, particularly for combating antibiotic-resistant skin infections, in the development of wound dressings and other healthcare materials.

## Graphical abstract

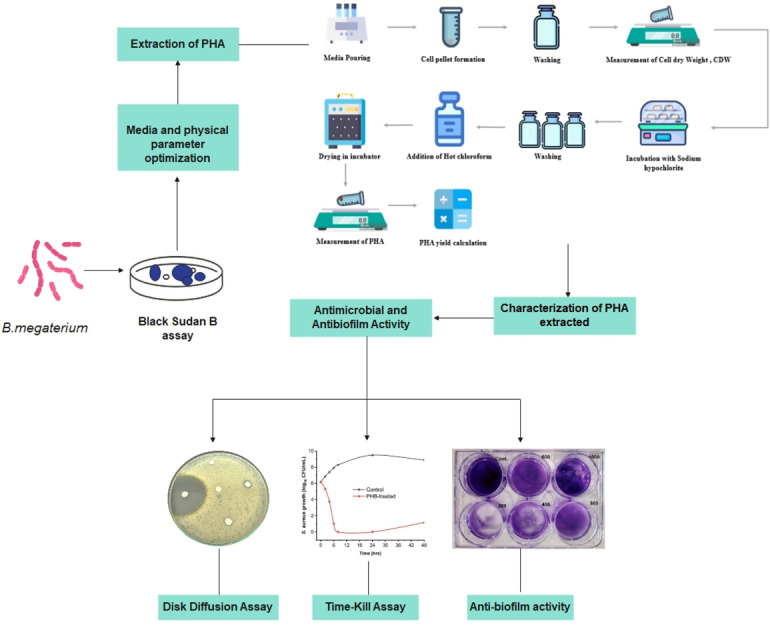

## 1 Introduction

The World Health Organization (WHO) recently reported that antimicrobial resistance AMR caused 1.27 million deaths in 2019, a staggering figure projected to increase to 10 million deaths annually by 2050 if left untreated.^1^ Skin infections caused by MRSA, XDR bacteria and extensively drug-resistant *Pseudomonas aeruginosa* are becoming more difficult to treat. In America, it is estimated that MRSA results in more than 100 000 severe cases every year, with about 20% ending in death. The cost to healthcare systems is huge, as the first year after a superbug infection with SSTIs adds $3 billion to treatment.^2^ Treating infections related to biofilms is more challenging because biofilms protect pathogens from the immune system and antibiotics. Many studies have revealed that more than 80% of chronic wound infections occur because of biofilm-forming bacteria that can resist very high doses of antibiotics.^3^ There is also an ongoing shortage of new antibiotics entering the market. There have been only a dozen antibiotics approved since 2017, and none directly targets growing health issues, such as MRSA or *Candida auris*, according to a 2023 World Health Organization report.^4^

Approximately 25% of chronic wound infections can be attributed to infectious bacteria, including *Staphylococcus epidermidis* and *Pseudomonas aeruginosa*. Almost half of the treatments fail because of efflux pumps and biofilm matrices found in many bacteria.^5^ We now observe that due to microbial resistance, we should consider PHA-based therapies because they act on multiple types of microbes, disrupt biofilms and may have less of a risk for resistance. An extra reason for failure is that biofilms and microspores retain 28% of the pathogenic microbes in the hospital, avoiding action from disinfecting chemicals. As a result, individuals with these illnesses must stay in the hospital over two and a half times longer than average, causing an increase of $3.2 billion in annual health costs in the U.S. (CDC, 2023). Four significant pathogens, including *S. aureus* and *K. pneumoniae*, were considered, with an estimated 670 000 deaths annually attributed to resistance against those pathogens in 2019.^6^

In Pakistan, in the past decade, various instances of resistant cases have been reported.^7^ For instance, an outbreak of XDR Salmonella showed complete resistance to fluoroquinolones.^8^ Likewise, a bloodstream infection study showed 93.7% resistance to third-generation cephalosporin.^9^ Skin diseases caused by pathogens are a significant health concern in Pakistan. A study conducted in a tertiary care hospital revealed that out of 300 patients, 23.3% were diagnosed with acne, making it the most frequent skin disease.^10^ Fungal infections were the most common among men, accounting for 21.2% of cases.^10^

With an increase in antimicrobial resistance (AMR), many traditional antibiotics and antiseptics have become ineffective, emphasizing the need for new therapeutic strategies. Conventional antimicrobials can pose risks due to toxicity, environmental persistence, and disruption of beneficial microbiota. Therefore, there is a crucial need for new sustainable methods that are both safe and effective.^11^ PHAs are considered promising candidates due to their antimicrobial properties, environmental degradability, and ability to complement other agents without inducing resistance.^12^

PHAs are produced by microorganisms and act as natural factories for these versatile types of biopolymers. When nitrogen or phosphorus is scarce, *Cupriavidus necator* and *Pseudomonas* species utilize PHAs for both energy supply and carbon storage.^13^ They employ enzymes such as PHB synthase, which is encoded by (PhaC), β-ketothiolase, which is encoded by (PhaA), and acetoacetyl-CoA reductase, which is encoded by (PhaB), and convert excess carbon into polyester granules.^14^ The method by which the strain converts carbon into PHB depends on the strain that produces scl-PHA, mcl-PHA, or copolymers of these PHA forms. CRISPR-Cas9 and other advances in microbial engineering have led to the enhanced production of PHB.^15^*C. necator* produced PHB with a yield of 44.9%.^16^ In addition, engineers have designed microbes to convert low-cost materials, such as lignocellulosic biomass, into functional PHAs with antimicrobial effects, especially when monomers, like 3-hydroxyvalerate, are incorporated. This demonstrates the potential of PHB for sustainable applications.^17^

Bacillus species are ideal for PHB production, yielding up to 85% PHB higher than many other bacteria and reproducing more rapidly.^18^ Because these biopolymers are GRAS-certified, they are suitable for medical use because they induce minimal inflammation. The market for Bacillus-based biopolymers is expected to grow at an annual rate of 8.9% from 2023 to 2030 (Grand View Research, 2023). This growth is expected to be propelled by investments from international companies, which are projected to generate 2.4 million tons by 2024.^19^ Researchers have found that Bacillus-derived PHAs are more effective against MRSA.^20^ During the microbial production of polyhydroxyalkanoates, different species of bacteria synthesize these polymers as internal stores of usable energy when nutrients are scarce. Among numerous species of microbes, we find that *Cupriavidus necator*, *Pseudomonas putida* and *Bacillus megaterium* produce PHB efficiently.^19^

The production of PHAs is managed through well-defined pathways, where different proteins work together to build materials using acetyl-CoA molecules. The microbial system used in synthesis offers benefits over traditional chemical methods, including mild operation, eco-friendly resources, and completely biocompatible polymers. For this reason, microbial PHAs are commonly used in biomedical products that must be free from contaminants. Generally, the structured synthesis of PHAs is developed through three steps:^1^ cultivating bacteria under normal conditions,^2^ causing PHB accumulation under nutrient stress (such as nitrogen or phosphorus shortage), and (ref. 3) separating and purifying intracellular granules.^21^

*Cupriavidus necator* stores PHB from carbon sources,^16^ while *Pseudomonas putida* uses PHAs in medical implants.^22^*Halomonas* spp. use PHB production in areas with very salty water,^23^ and *Haloferax mediterranei* can produce PHAs by processing waste rich in starch.^24^ Even with all the different species, *Bacillus* remains the preferred microbe for clinical work because it is Generally Recognized As Safe (GRAS) and can be safely modified through genomics.^25^

Two different ways in which polyhydroxyalkanoates deviate from the usual resistance mechanisms are crucial for their success against microbes. Initially, they interfere with membranes through hydrophobic binding and are unrelated to typical molecular targets. In the second instance, acidic compounds, such as 3-hydroxybutyrate, build an antimicrobial microenvironment that depends on acidity.^26^ Additionally, engineered nanoparticle preparations can penetrate deeply into the structure of biofilms. PHA coatings work even after they dry up, unlike regular antiseptics, and have been found in recent clinical trials to reduce hospital surface contamination rates by up to 89% over a period of 3 to 4 days.^27^ Worldwide, the use of current disinfectant technologies generates approximately 580 000 tons of persistent waste annually, according to the UNEP (2023). It was determined that PHB solutions reduce the carbon impact of infection control by 76% because they are made from renewable materials and are fully broken down after use.^12^ PHAs do not form dangerous byproducts, unlike chlorinated disinfectants, which tend to do so; therefore, they are more environmentally friendly and can help address major health concerns associated with the current methods of disinfection (EPA, 2023). Because they offer environmental benefits, PHAs become key in helping protect healthcare and many communities from infections.^12^ The primary aim of this study is to optimize, extract, purify, and characterize the polyhydroxyalkanoates (PHAs) produced, followed by their antimicrobial and antibiofilm activities. PHAs produced by *Bacillus species* offer significant advantages in healthcare due to their biocompatibility, environmental sustainability, and potential antimicrobial and antibiofilm properties.

Dealing with *Staphylococcus aureus*, *Staphylococcus epidermidis*, and *Candida albicans* is especially crucial in the context of antimicrobial resistance because these pathogens have shown an increasing ability to resist common antimicrobial agents. Over time, the widespread use of antiseptics in both healthcare and community settings has led to the development of resistance in these microorganisms. *Staphylococcus aureus* and *Staphylococcus epidermidis*, particularly strains resistant to antibiotics, such as methicillin (MRSA), can also exhibit resistance to antiseptic agents, including chlorhexidine and alcohol.^28^ Similarly, *Candida albicans* can develop resistance to antifungal agents, complicating treatment efforts.^29^ The rise in antiseptic resistance means that traditional methods of infection control and prevention may no longer be as effective, making it more difficult to manage outbreaks and hospital-acquired infections.^30^ This highlights the importance of exploring alternative methods, enhancing infection control practices, and adopting targeted approaches to prevent the development of further resistance.^31^ Addressing antiseptic resistance is essential not only to preserve the effectiveness of existing treatments but also to ensure better patient outcomes, especially in vulnerable populations, like those with compromised immune systems or medical devices.^31^ Polyhydroxyalkanoates are a huge class in this regard. PHB has been of interest in recent times due to its properties, such as biodegradability, thermal stability, bioavailability, and sustained action.^32^ Due to the increasing prevalence of antibiotic resistance, treatment and sanitation issues are escalating in healthcare settings. Additionally, bacterial and fungal infections, particularly those related to the skin, have become increasingly difficult to treat. This study aims to provide an alternative option that is biodegradable and develops slower resistance. The primary target was to optimize the bacterial growth conditions for maximum PHB accumulation in the bacterial cells, followed by successful extraction and purification. The extracted PHB from a bacterial source was then evaluated for its antimicrobial and antibiofilm effects. This integrated approach provides an environmentally friendly route for PHB production and explores PHAs as promising alternatives to conventional antiseptics, thereby addressing the dual challenge of rising antiseptic resistance and environmental sustainability, which aligns well with the Sustainable Development Goals, SDG-3 and SDG-12.

## 2 Methodology

### 2.1 Collection of the PHA-producing bacterial sample and its characterization

The bacterial sample *Bacillus megaterium* (NCCP-455)*,* used for PHB production (isolated from soil), was retrieved from NCCP and NARC. The sample was immediately shifted to the microbiology laboratory of the National University of Medical Sciences and stored at 4 °C before further processing. The sample was streaked on TSA (Tryptic Soy Agar, Bioworld) plates, and a series of subcultures were carried out to refresh the bacterial strain using a standard protocol (Kojuri *et al.*, 2021).^33^ The samples were also preserved at −80 °C as glycerol stocks for future use. PHA production was initially confirmed using the method followed by.^34^ The bacterial isolate was initially chosen for qualitative analysis using Sudan Black B stain.^35^

### 2.2 Media preparation and optimization for the production of PHAs

Prior to the inoculation of *B. megaterium* for PHB production, a seed culture with viable and fresh bacterial cells was prepared. Initially, the broth used for seed culture was of three types: nutrient broth (Sigma-Aldrich), tryptic soy broth (TSB; Bioworld), and modified nutrient broth (Sigma-Aldrich: supplemented with yeast extract, beef extract, peptone and sodium chloride (NaCl), kept for 37 °C in a shaking incubator at 120 rpm for 18–24 h). With maximum OD values obtained from TSB, it was selected as the seed culture medium. 5% of the seed culture was used as the initial inoculum to produce PHA. PHB production is best achieved when the bacterial sample is supplemented with a carbon-rich, nitrogen- and phosphorus-limited environment. For this, a medium with certain modifications was used. The modified minimal salt medium broth was created according to the protocol used by.^36^ (MM1) was created with 5 major components: ammonium sulphate (NH_4_)_2_SO_4_, 2 g L^−1^; potassium dihydrogen orthophosphate (KH_2_PO_4_), 13.3 g L^−1^; magnesium sulphate heptahydrate (MgSO_4_·7H_2_O), 0.45 g L^−1^; citric acid (C_6_H_8_O_7_), 1.7 g L^−1^; and carbon source (glucose (C_6_H_12_O_6_)/glycerol (C_3_H_8_O_3_)), 2%. To this was added a10 ml L^−1^ trace element solution formed using 9 constituents: Iron(ii) sulphate heptahydrate (FeSO_4_·7H_2_O), 10 g L^−1^; zinc sulphate heptahydrate (ZnSO_4_·7H_2_O), 2.25 g L^−1^; copper(ii) sulphate pentahydrate (CuSO_4_·5H_2_O), 1 g L^−1^; calcium chloride (CaCl_2_), 2 g L^−1^; manganese(ii) chloride (MnCl_2_), 0.05 g L^−1^; and sodium molybdate dihydrate (Na_2_MoO_4_·2H_2_O), 0.1 g L^−1^ 1 M boric acid (H_3_BO_3_) (4.75 mL L^−1^) and 1 M sodium hydroxide (NaOH) (2.38 mL L^−1^) were used for the *in situ* preparation of sodium tetraborate (Na_2_B_4_O_7_)/borax, and 35% hydrochloride (HCl) (10 mL L^−1^) was also added in the trace element solution.

After an overnight incubation of *B. megaterium* in tryptic soy broth (Bioworld) in a shaking incubator at 120 rpm for 18–24 h, the mixture was transferred 5% to the modified MSM (MM1), 500 mL of media each. The Duran bottles used were covered with tissue culture plastic sealing film, which had filter paper with a pore size of 0.2 μm, allowing for the smooth flow of oxygen and carbon dioxide necessary for bacterial growth. The media was initially incubated at 50 rpm for an hour; then, the speed was increased to 120 rpm. The temperature was set to 37 °C, and the time of incubation ranged from 64 to 72 h. Modified MSM (MM1) without the bacterial inoculum was considered the negative control. This was then subjected to extraction.

An orthogonal experimental design (OED) was employed to systematically investigate the influence of multiple factors on PHB production by *Bacillus megaterium*. This statistical approach was selected due to its ability to efficiently evaluate the effects of several variables and their interactions with a reduced number of experimental runs compared to traditional one-factor-at-a-time methods. The factors investigated included carbon source (glucose, glycerol, and sucrose), nitrogen source (ammonium sulphate, peptone, and yeast extract), incubation time (48, 64, and 72 h), agitation speed (0, 60, and 120 rpm), citric acid (1 g L^−1^, 2 g L^−1^, 3 g L^−1^ and 4 g L^−1^), NaCl stress (7%, 10% and 13%) and inoculum age (12, 18, and 24 h). The experimental setup was created using SPSS software (version 25.0, IBM, USA). The orthogonal design was particularly advantageous in this study because it not only reduced the experimental workload but also provided insights into potential interactions between factors that could influence PHB biosynthesis. The protocol followed was a modified form of.^37^

### 2.3 Extraction assay for PHAs

The extraction of PHB was performed using the hypochlorite method described by^38^ with a few modifications. The solvent extraction method using chloroform coupled with the sodium hypochlorite method was used.^39^ The modified MSM media was subjected to centrifugation. Specifically designed 50 mL centrifuge tubes (LABTron centrifuge) with settings of temperature at 4 °C, speed of 12 000 rpm, and a cycle of 10 min were applied. The first round yielded a white, grayish pellet containing the *B. megaterium* cells. The supernatant was discarded, and the pellet was resuspended in PBS (Phosphate Buffer Saline, pH 7.5) and centrifuged again to remove any impurities. The cell dry weight was recorded. A 1 : 1 volume of sodium hypochlorite (NaOCl) was added; then, the mixture was incubated at 37 °C in a shaking incubator at 120 rpm for 90 min. Sodium hypochlorite lyses the cells, thereby releasing the PHB granules. This solution was then centrifuged again, leaving behind the pellet containing the PHA. This was then thoroughly washed with distilled water, methanol, and acetone to remove any remaining impurities. The final pellet was then suspended in an equal volume of 1 : 1 hot chloroform and then left to dry at 40 °C. The weight of the extracted pellet was calculated, and the results were recorded using the formulas;
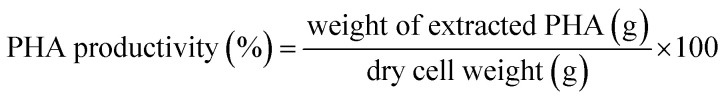

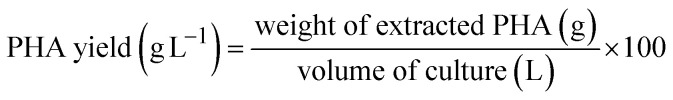


### 2.4 Characterization of PHAs

The PHAs extracted from various samples were subjected to Fourier Transform Infrared Spectroscopy (FTIR) to identify functional groups in PHB using a well-established protocol^40^ with minor modifications. For this, a small amount of dry PHA, about 5 mg, was used to prepare a thin film by dissolving the polymer in an organic solvent, chloroform, due to its lipophilicity and allowing it to evaporate naturally on a clean glass slide film or a KBr disc. The FTIR spectra were then finally recorded in the range of 4000–500 cm^−1^ using an FTIR spectrometer.

Further, H^−1^-NMR and C^−13^ NMR spectra of PHB samples were obtained commercially^41,42^ to determine the carbon skeleton and monomer composition of the extracted PHA. 5–10vmg approx. A sample of PHB was taken and dissolved in 0.6 mL of deuterated chloroform (CDCl3). This led to the formation of a clear solution, which was then transferred to a 5 mm clean NMR tube and subjected to analysis using a 400 MHz NMR spectrometer at 25 °C, and the spectra were recorded.

The thermal stability of the extracted PHB was evaluated and compared with that of a commercial standard PHB using thermogravimetric analysis (TGA). The analysis was conducted at a temperature ranging from 30 °C to 600 °C at a constant heating rate of 10 °C min^−1^ under a nitrogen atmosphere (N_2_ flow rate: 40 mL min^−1^) to prevent oxidative degradation. The thermal properties of PHB were analyzed by DSC^43^ using hermetically sealed aluminium pans under a nitrogen purge of 50 mL min^−1^. Samples (5 mg) were vacuum-dried at 40 °C and subjected to a heat–cool–reheat cycle, heating from 0 to 300 °C at 10 °C min^−1^. The second heating curve was used to determine the glass transition temperature (*T*_g_), melting temperature (*T*_m_), and crystallization temperature (*T*_c_). The nature of PHB was analyzed using X-ray diffraction^44^ with a diffractometer (GNR explorer X-ray diffractogram m2021). Samples were scanned over a 2*θ* of 5°–60° with a step size of 0.02° and a scan rate of 2° min^−1^.

### 2.5 Enrichment of skin-related pathogens

Skin-related pathogens were retrieved from the University of Veterinary and Animal Sciences, Lahore. The provided bacterial and fungal strains included (*Staphylococcus aureus*, *Staphylococcus epidermidis*) and *Candida albicans.* They were then streaked on their respective media: mannitol salt agar (Bioworld) for *S. aureus* and *S. epidermidis*, and Sabouraud dextrose agar (Bioworld); they were also preserved at −80 °C as glycerol stocks for further use. This is similar to the protocols mentioned by.^45^

### 2.6 Antimicrobial activity of PHB

The agar disc diffusion method^46^ was applied to assess the zone of inhibition around PHB samples placed on an agar plate inoculated with bacteria. MH agar plates were used for the disk. Overnight microbial cultures after adjusting the OD to 0.3 were used. PHB was first dissolved in chloroform as a stock solution, followed by the loading of PHB onto the discs. The discs were left to rest for 10 minutes to allow the chloroform to evaporate. For the control, 10 μL of chloroform was also applied to one of the discs as a negative control. The plates were finally placed in an incubator for 18–24 h at 37 °C, and the zones were measured.

The microdilution method was used to determine the minimum inhibitory concentration (MIC) of PHB by applying the method of.^47^ A 96-well sterile microtiter plate was used, following the 2024 guidelines of the Clinical and Laboratory Standards Institute (CLSI) with certain modifications. The stock solution of PHB was prepared in chloroform/DMSO with known PHB concentrations. Serial dilutions of PHB stock were prepared in MH broth (Mueller Hinton, Bioworld). Bacterial cultures were grown overnight and adjusted to 0.5 McFarland standard before being poured into the wells. Each well received a total volume of 200 μL, consisting of 100 μL of the diluted PHB solution and 100 μL of the bacterial suspension. Three controls were also set: media only (negative/sterility control), bacteria without PHB (positive/growth control), and chloroform/DMSO without PHB but bacteria (solvent control) to rule out solvent effects. Plates were then incubated at 37 °C for 18–24 hours, and the optical density was measured at 600 nm using a microplate reader. MIC is considered the lowest concentration of PHB that inhibits the visible growth of bacteria.

### 2.7 Time-kill assay

The time kill assay was used to determine how quickly PHB inhibits or kills bacteria over a specified period. For this, the framework of^46^ is used. PHB's antibacterial effect was assessed against bacteria over time using MIC concentrations. 5 μL of PHB at its MIC value was added to 15 mL of MH broth with a bacterial colony to study the effect of PHB on bacterial growth and left in a shaking incubator at 37 °C and 120 rpm. Bacterial growth was observed every two hours, starting from 0 h to 8 h. A control was used where no PHB was added. After every interval, the CFU count was done by swabbing 100 μL of bacterial solution on MH agar plates and incubating at 37 °C for 24 h. A positive control was used where no PHB was added; bacteria continued to grow, and the negative control was broth only.

### 2.8 Antibiofilm activity of the extracted PHB

Antibiofilm activity was analysed in two aspects: pre-treatment/inhibition of biofilm with PHB using the protocol followed by^48^ and the protocol^49^ for the eradication of formed biofilm using PHB with certain modifications.

#### 2.8.1 Inhibition of biofilm using PHB

To assess the ability of the extracted PHB to inhibit biofilm, a 6-well sterile plate was used. PHB was dissolved in 2–3% w/v chloroform/DMSO, and 1 mL was transferred to each well. The solvent was PHB was able to form a thin film at the bottom of the well. Wells without PHB coating served as negative controls. The bacterial culture was prepared in nutrient broth (Sigma-Aldrich) and set to a 0.5 McFarland standard. A 1 : 100 dilution of this bacterial culture in nutrient broth with a volume of 3 mL was poured in each well (both types: PHB coated and positive control) and left for incubation at 37 °C for 24–48 h statically. The plate was gently rinsed three times with PBS to remove any non-adherent cells. The remaining attached cells of the biofilm were stained with 0.1% crystal violet for 20 minutes and washed again to remove excess dye. The retained dye was solubilized using 33% glacial acetic acid/ethanol, and the absorbance was measured at 570 nm.

#### 2.8.2 Eradication of biofilm using PHB

For biofilm eradication, the formation of mature biofilm prior to PHB inoculation was performed. For this, bacterial cultures set to 0.5 McFarland standard were used. A 1 : 100 dilution of this bacterial culture in nutrient broth with a volume of 3 mL was poured into each well of a 6-well sterile plate and left for incubation at 37 °C statically for 24–72 h. After incubation, the plates were washed gently with PBS to remove the planktonic cells. Freshly prepared PHB solutions with known concentrations (100–1000 μg mL^−1^) in chloroform/DMSO were prepared, and 3 mL of these solutions were added to the wells. Broth and bacteria were used as positive controls. The plate was then statically incubated at 37 °C for 24 h. Washing with PBS was done three times. The remaining attached cells of the biofilm were stained with 0.1% crystal violet for 20 minutes and washed again. The retained dye was solubilized using 33% glacial acetic acid/ethanol, and the absorbance was measured at 570 nm.

#### 2.8.3 Assessment of biofilm inhibition and eradication using cell viability assay/XTT assay

To assess the cell viability of the biofilm in the case of inhibition, the protocol of^50^ was used with slight modifications. A 96-well sterile plate was used in which PHB was dissolved in 2–3% w/v, and 100 μL of this solution was transferred to each well. The solvent was evaporated, and a thin film was formed at the bottom of the well. Wells without PHB coating served as negative controls. The bacterial culture set to 0.5 McFarland standard was used. A 1 : 100 dilution of this bacterial culture in nutrient broth with a volume of 200 μL was poured into each well (both types: PHB coated and positive control). The plate was left for incubation at 37 °C for 24–48 h statically. The plate was gently rinsed three times with PBS (Phosphate Buffer Saline with pH 7.5) to remove any non-adherent cells. The metabolic activity of the remaining attached cells of the biofilm (viable biomass) was assessed by adding 0.5 mg mL^−1^ XTT in PBS (Phosphate Buffered Saline) freshly activated with 1 μM menadione into each well. Plates were then incubated in the dark for 2–3 h at 37 °C. The resulting orange formazan compound was carefully transferred to a cuvette, and the readings were taken at 490 nm using a spectrophotometer.

To assess the cell viability of the biofilm in the case of eradication, the same protocol^50^ was used with slight modifications. The formation of mature biofilm prior to PHB inoculation was done using a microbial culture set to 0.5 McFarland. A 1 : 100 dilution of this bacterial culture in nutrient broth with a volume of 200 μL was poured into each well of a 96-well sterile plate and left for incubation at 37 °C statically for 24–72 h. After incubation, the plates were gently washed with PBS to remove the planktonic cells. Freshly prepared PHB solutions with known concentrations (200–1000 μg mL^−1^) in chloroform/DMSO were prepared. 200 μL of these solutions was added to the wells. Bacteria and broth were used as positive controls. The plate was then statically incubated at 37 °C for 24 h, followed by washing three times with PBS. The metabolic activity of the remaining attached cells of the biofilm (viable biomass) was assessed by adding 100 μL of 0.5 mg mL^−1^ XTT in PBS freshly activated with 1 μM menadione into each well. Plates were then incubated in the dark for 2–3 h at 37 °C. The resulting orange formazan compound was carefully transferred to a cuvette, and readings were taken at 490 nm using a spectrophotometer.

### 3. Results

#### 3.1 Sample collection of PHA-producing bacteria and their confirmation

**Fig. 1:**
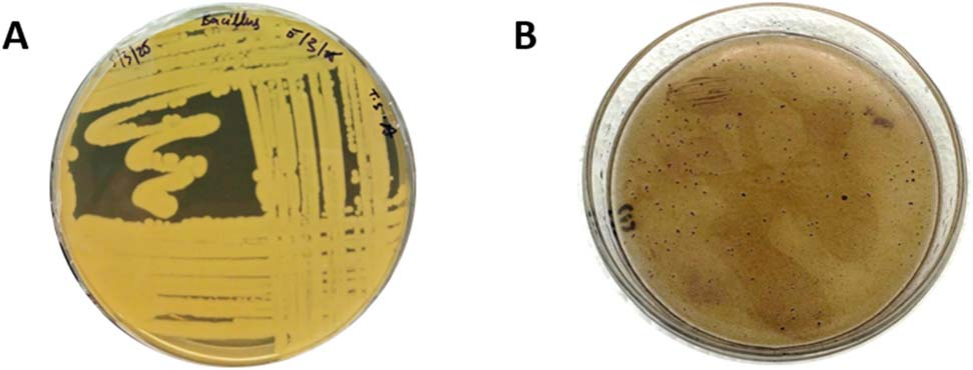
(A) *Bacillus megaterium* on TSA plates. (B) PHA accumulation showing intensity (++++) using a Black Sudan (B) plate assay.

The isolated sample was streaked on TSA plates and confirmed for PHB production, yielding bluish colonies on MSM plates using a Back Sudan B plate assay. The dark color shows good PHB accumulation in *B. megaterium* cells, as shown in Fig. 1.

### 3.2 Media preparation for the production of PHAs

The OED identified ammonium sulphate (2 g L^−1^) + glucose + 120 rpm agitation + 10% NaCl + 67 h incubation (Condition 8) as the optimal combination, yielding the highest PHB productivity (46.7%), as shown in Table 1. The design's ability to decouple confounding variables revealed that citric acid (2–4 g L^−1^) plays a critical role in enhancing acetyl-CoA flux toward PHB synthesis while maintaining cellular growth. In contrast, sodium nitrate and peptone as nitrogen sources generally result in lower yields (0.08–0.25 g L^−1^), indicating that nitrogen availability and assimilation efficiency play a crucial role in PHB biosynthesis. Glucose consistently outperformed glycerol and sucrose as a carbon source likely due to its faster metabolization. Additionally, higher agitation speeds (120 rpm) correlated with improved PHB production possibly due to enhanced oxygen transfer, while static conditions (0 rpm) resulted in reduced yields (Card ID 2: 0.1 g L^−1^).

**Table 1:** Orthogonal experimental design variations, yield and productivity

Card ID	Nitrogen source	Citric acid	Carbon sources	Agitation speed	Inoculum age	NaCl stress	Incubation time	PHA yield (g L^−1^)	PHA productivity (%)
1	Sodium nitrate	2 g L^−1^	Glucose	120 rpm	18 h	7%	72 h	0.25	30.2
2	Ammonium sulphate	1 g L^−1^	Glucose	0	18 h	7%	24 h	0.1	12.5
3	Ammonium sulphate	4 g L^−1^	Glucose	60 rpm	20 h	7%	72 h	0.3	35
4	Yeast extract	2 g L^−1^	Glycerol	0	24 h	7%	72 h	0.18	22.1
5	Ammonium sulphate	4 g L^−1^	Sucrose	120 rpm	18 h	13%	48 h	0.22	27.8
6	Sodium nitrate	3 g L^−1^	Glycerol	0	18 h	10%	67 h	0.2	25
7	Peptone	4 g L^−1^	Glucose	0	24 h	10%	48 h	0.15	18.3
8	Ammonium sulphate	2 g L^−1^	Glucose	120 rpm	18 h	10%	67 h	0.4	46.7
9	Yeast extract	3 g L^−1^	Glucose	120 rpm	24 h	13%	67 h	0.35	40.5
10	Peptone	1 g L^−1^	Sucrose	120 rpm	24 h	7%	24 h	0.12	15
11	Ammonium sulphate	2 g L^−1^	Glycerol	120 rpm	18 h	10%	24 h	0.28	32.6
12	Yeast extract	4 g L^−1^	Glucose	0	18 h	10%	24 h	0.1	12
13	Peptone	3 g L^−1^	Glycerol	120 rpm	20 h	7%	48 h	0.32	38.1
14	Peptone	2 g L^−1^	Sucrose	0	18 h	7%	67 h	0.14	17.2
15	Sodium nitrate	1 g L^−1^	Glucose	0	24 h	7%	48 h	0.08	10
16	Peptone	4 g L^−1^	Glycerol	0	18 h	7%	72 h	0.17	20.8
17	Ammonium sulphate	3 g L^−1^	Sucrose	0	24 h	10%	72 h	0.23	28.9
18	Peptone	2 g L^−1^	Glucose	0	20 h	13%	24 h	0.09	11
19	Peptone	3 g L^−1^	Glucose	60 rpm	18 h	13%	72 h	0.27	31.7
20	Sodium nitrate	4 g L^−1^	Glycerol	60 rpm	24 h	13%	24 h	0.21	25.6

**Fig. 2:**
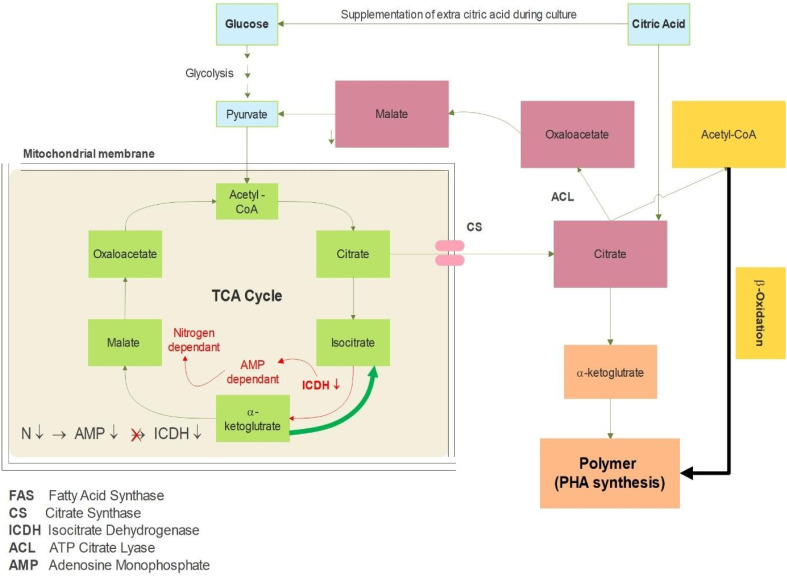
Interlink of different pathways and how citric acid plays a role in increased PHA accumulation.

NaCl stress exhibited a dose-dependent effect on PHB yield, with 10% NaCl proving optimal (Card ID 8: 0.4 g L^−1^), while extreme salinity (13%) reduced productivity in some cases (Card ID 5: 0.22 g L^−1^). A longer incubation period (67 h) generally supported higher PHB accumulation likely due to extended polymer synthesis during the stationary phase. However, shorter incubation times (24 hours) resulted in a diminished yield (Card ID 10: 0.12 g L^−1^), suggesting that the time was insufficient for PHB granule formation. The inoculum age also influenced productivity, with 18-hour cultures (mid-log phase) performing better than older inocula (24 h), possibly due to higher metabolic activity. Interestingly, citric acid supplementation (2 g L^−1^) improved PHB yields when paired with ammonium sulfate (0.3 g L^−1^) possibly by enhancing precursor availability in the TCA cycle. The trends highlight that nitrogen sources, carbon substrates, agitation and stress conditions are critical for maximizing PHB production in *Bacillus* spp. Moreover, from the results, we conclude that the additional supplementation of citric acid plays a crucial role in the increased production of PHB up to a certain level, as shown in Fig. 2.

### 3.3 Extraction assay for PHAs

**Fig. 3:**
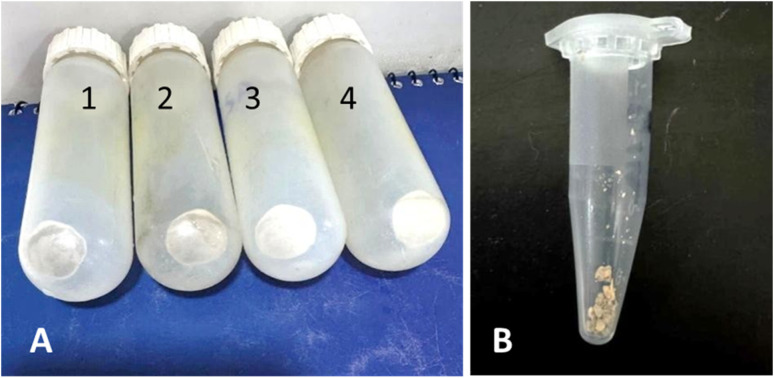
Cell pellets containing PHA. (A) Two types of cell pellets were formed. The tubes labelled 1 and 2 contained a greyish white pellet formed when glycerol was used as a carbon source. The tubes labelled 3 and 4 had white pellets. In this case, the carbon source used was glucose. (B) Final powdered form of PHA after treatment with hot chloroform at 40 °C. After the complete removal of chloroform, the weight of the powdered PHA was calculated.

The extraction of PHB was done using the sodium hypochlorite method, followed by solvent purification using chloroform. Initially, the MSM broth was taken out of the shaking incubator after 67 h, resulting in a maximum PHB yield obtained through this procedure, ultimately 0.4g L^−1^, which in terms of productivity can be explained as 46.7%. This is also shown in Table 1. One important observation during the extraction process was that the use of glycerol instead of glucose had no effect on the dry cell weight (DCW). The results demonstrate that even though the cell dry weight using both carbon sources was equal, more PHB accumulation was observed in the case of glucose as a carbon source. The PHB extracted was in powdered form with a sand-like colour, as shown in Fig. 3.
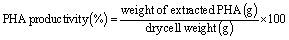

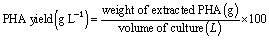


### 3.4 Characterization of PHAs

#### 3.4.1 Fourier-transform infrared spectroscopy analysis

**Fig. 4:**
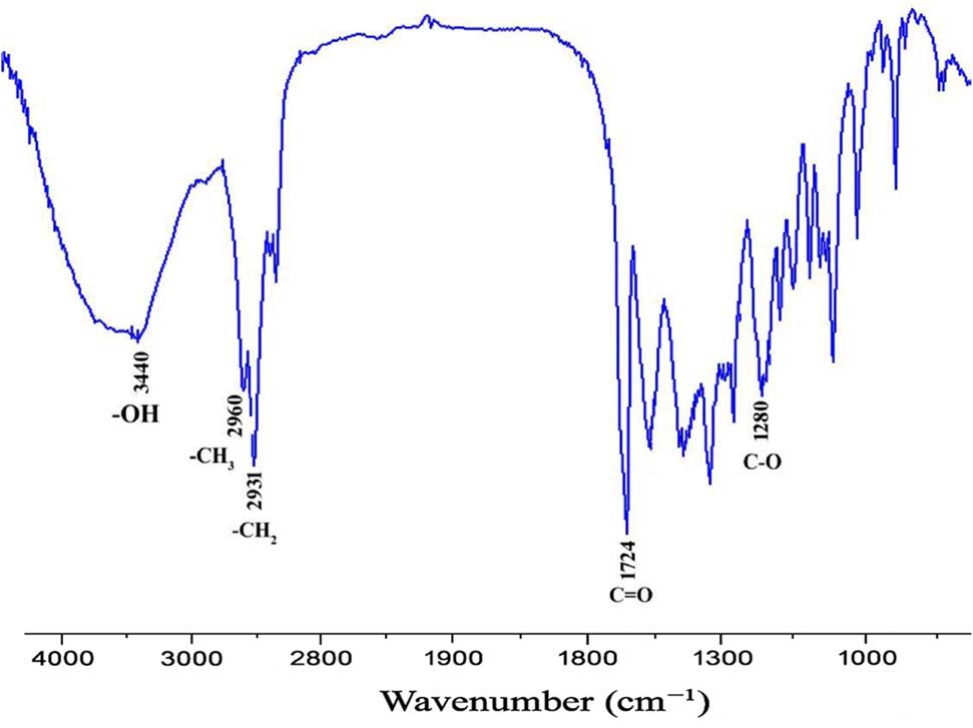
Fourier-Transform infrared spectroscopy characterization of PHA extracted from *Bacillus megaterium.*

The structural identity and functional group composition of the extracted polymer were determined using Fourier-transform infrared (FTIR) spectroscopy. The most intense peak appeared around 1720 cm^−1^, indicating the stretching vibration of the carbonyl (C=O) group in the ester linkage of PHB. Another significant absorption band was observed near 1278 cm^−1^, representing the C–O stretching vibration of the ester bond. Additional peaks in the range of 1050–1300 cm^−1^ were identified, corresponding to various C–O–C and C–C stretching modes. These peaks collectively confirm the presence of an aliphatic polyester backbone typical of PHB. The spectrum also showed a series of absorption bands associated with the aliphatic –CH_2_ and –CH_3_ groups. The spectral pattern closely matches the reference spectra for standard PHB reported in the existing literature. The strength of the carbonyl peak at 1720 cm^−1^ was particularly notable, indicating a high degree of ester linkage, which can be indirectly linked to the polymer's crystallinity and molecular integrity, as shown in Fig. 4.

#### 3.4.2 Nuclear magnetic resonance (NMR)

**Fig. 5:**
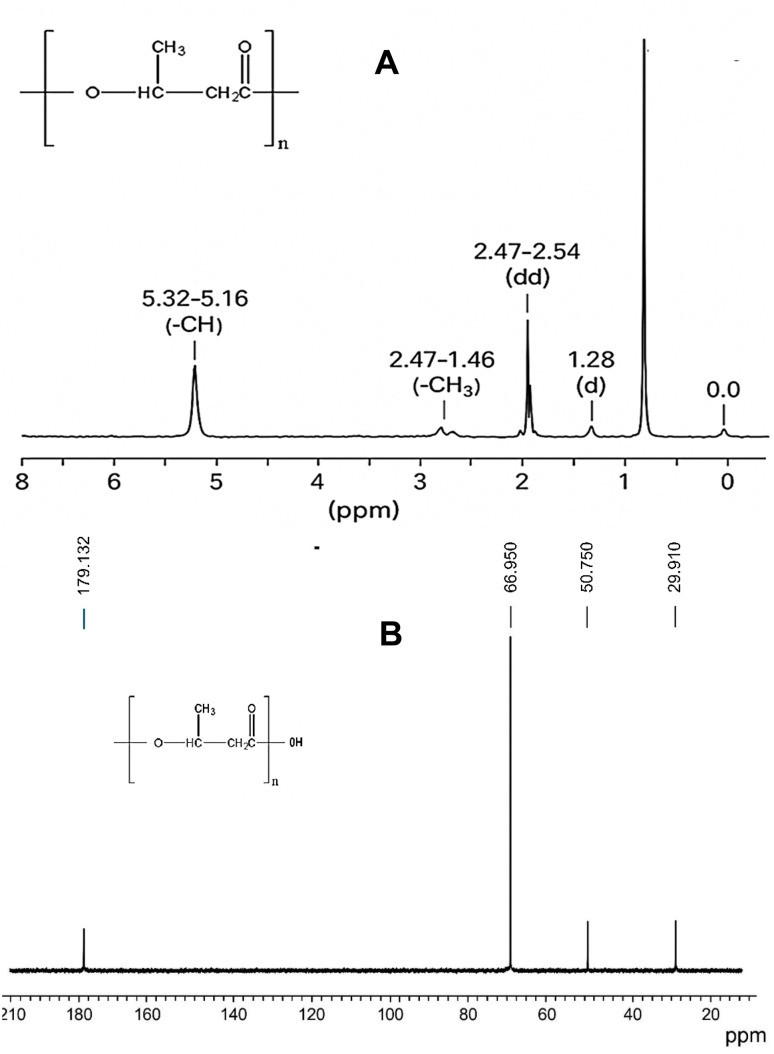
(A) NMR spectral analysis of the extracted PHB. (A) ^1^H NMR spectrum showing characteristic peaks for methyl (CH_3_), methylene (CH_2_), and methine (CH) groups. (B) ^13^C NMR spectrum confirming the presence of carbonyl (C=O), CH, CH_2_, and CH_3_ carbon signals.

The NMR spectrum (Fig. 5) revealed prominent resonance signals at chemical shifts (*δ*) of approximately 5.2 ppm, 2.5 ppm, and 1.2 ppm. These peaks are characteristic of the structural protons found in PHB. Specifically, the resonance at *δ* ≈ 5.2 ppm is attributed to the methine proton (–CH–) located adjacent to the ester functional group within the PHB monomeric unit. The multiplet observed at *δ* ≈ 2.5 ppm corresponds to the methylene group (–CH_2_^−^) situated next to the carbonyl carbon, while the doublet near *δ* ≈ 1.2 ppm reflects the terminal methyl group (–CH_3_) in the polymer's repeating structure, as shown in Fig. 5a. Notably, the chemical shifts recorded in our analysis corresponded well with the reference values reported in the established literature, underscoring the structural fidelity of the PHB polymer produced in our system. ^13^C NMR spectrum (Fig. 5b) exhibited additional structural confirmation. The spectrum showed characteristic carbon signals for PHB: ∼169.0 ppm for carbonyl carbon (C=O), ∼67.0 ppm for methine carbon (CH), ∼40.8 ppm for methylene carbon (CH_2_), and ∼19.7 ppm for methyl carbon (CH_3_). These values are consistent with previously reported standard PHB spectra.^42^

#### 3.4.3 Thermogravimetric analysis

**Fig. 6:**
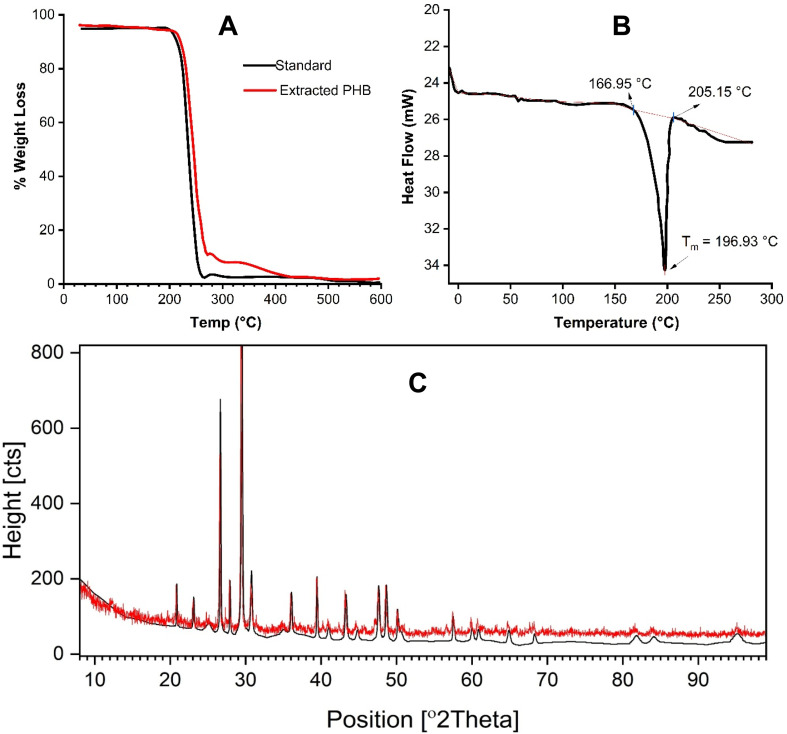
(A) Thermogravimetric (TGA), (B) differential scanning calorimetric (DSC), and (C) X-ray diffraction (XRD) characterization of the extracted and purified PHB.

The thermal stability of PHB was assessed using thermogravimetric analysis (TGA), as shown in Fig. 6a. The TGA curves revealed a two-step weight loss pattern for all samples, including the standard PHB and the PHB extracted from *B. megaterium*. The first stage of weight loss occurred between 100 °C and 160 °C, with a minor reduction of about 1.2%, attributed to the evaporation of residual solvents, such as chloroform or methanol, trapped in the polymer matrix. This initial drop was consistent across all samples. The major thermal degradation began after 180 °C, following the polymer's melting point. This phase involved a rapid loss of mass due to the breakdown of the PHB chains. The degradation likely occurred through random chain scission, especially at the ester linkages, along with hydrolytic degradation. These changes are common during the thermal decomposition of PHB and contribute to the formation of low molecular weight by-products, like crotonic acid. The maximum degradation temperature (*T*_max_) of PHB from *B. megaterium* was observed at 415 °C, which is significantly higher than that of the commercial PHB standard. This suggests improved thermal resistance.

#### 3.4.4 Differential scanning calorimetry (DSC)

The thermal transitions of the PHB samples were analyzed using differential scanning calorimetry, and the resulting heating and cooling curves are presented in Fig. 6b. This analysis helped evaluate key thermal properties, including melting temperature (*T*_m_), glass transition temperature (*T*_g_), cold crystallization temperature (*T*_c_), and degree of crystallinity (*X*_c_). During the first heating cycle, PHB synthesized from *B. megaterium* exhibited melting behavior similar to that of the standard. The melting temperature (167 °C) is consistent with values reported in previous studies.^33^ In contrast, the glass transition temperature varied notably from the standard. The PHB from *B. megaterium* showed a higher *T*_g_ value (up to 6 °C), while the commercial standard exhibited a lower *T*_g_ value of around −8 °C. This variation may be attributed to differences in polymer chain mobility and internal structure. The more amorphous nature of the bacterial PHBs likely restricts chain movement, resulting in elevated *T*_g_ values. These findings are comparable to previously published data on PHB derived from similar microbial sources. The degree of crystallinity (*X*_c_) was calculated based on the enthalpy of fusion using 146 J g^−1^ as the enthalpy of 100% crystalline PHB. PHB produced by *B. megaterium* displayed a crystallinity of 44%, which was lower than that typically reported for pure crystalline PHB, suggesting a less rigid polymer structure. This reduced crystallinity may enhance flexibility and broaden the material's application potential by decreasing brittleness.

#### 3.4.5 X-ray diffraction (XRD) analysis

The XRD pattern presented in Fig. 6C displays distinct diffraction peaks across a wide 2*θ* range (10°–90°), indicating the crystalline nature of the PHB sample. Prominent and sharp peaks are observed around 27°, 31°, and 45° (2*θ*), with the most intense peak located near 31°, suggesting a highly ordered crystalline structure. The multiplicity and sharpness of these peaks reflect the presence of well-defined crystalline domains, and the absence of broad humps suggests minimal amorphous content. The high intensity at certain positions further supports a preferential orientation of specific crystallographic planes within the sample.

The red and black patterns represent the extracted PHB (red) and reference (black). The close alignment between them confirms that the synthesized material shares a high degree of structural similarity with the standard reference material. These are the most intense reflections and are typically associated with the orthorhombic unit cell structure of PHB. The diffraction patterns in this study are consistent with the literature values, confirming that both bacterial strains produce PHB with typical semi-crystalline characteristics. The structural features observed are also aligned with the known helical arrangement of polymer chains in crystalline domains.

### 3.5 Enrichment of skin-related pathogens

**Fig. 7:**
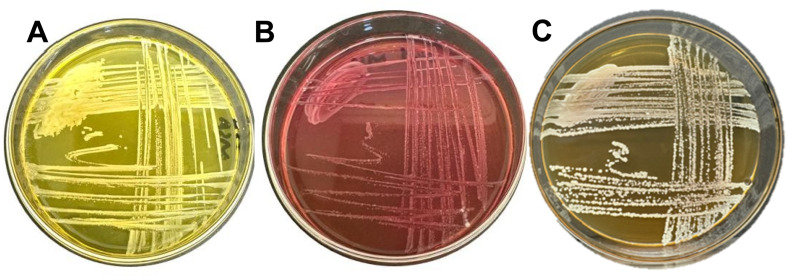
Growth of *Staphylococcus aureus* (A) and *Staphylococcus epidermidis* (B) on differential media mannitol salt agar (MSA), and *C. albicans* on its selective media Sabouraud dextrose agar (SDA).

The enrichment of *S. aureus*, *S. epidermidis* and *C. albicans* was done on their respective media (Fig. 7). For *S. aureus* and *S. epidermidis*, mannitol salt agar was used, which was both selective and differential. The media is originally pink in colour. *S epidermidis* retains the pink colour, while *S. aureus* ferments the mannitol, leading to a yellow colour due to a change in pH. *S. aureus* showed golden, round colonies with a glossy appearance, while *S. epidermidis* showed opaque colonies with a slightly dry effect. In the case of *C. albicans*, a common fungal medium, Sabouraud Dextrose Agar, was used. The fungal colonies of *C. albicans* observed were off-white, round, and slightly moist, with a yeast-like appearance.

### 3.6 Antimicrobial activity of PHB

**Fig. 8:**
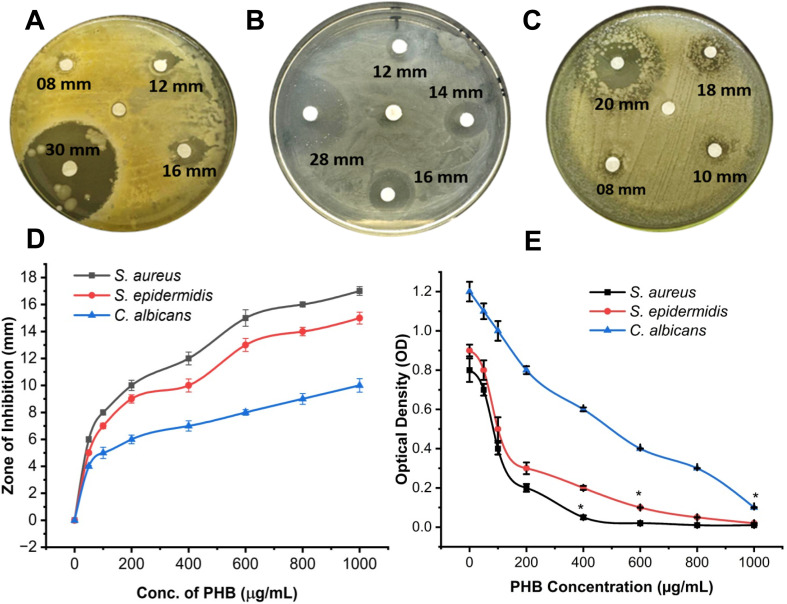
Antibacterial activity of different concentrations (100, 400, 600, and 1000 μg mL^−1^, respectively) of PHB against (A) *S. aureus*, (B) *S epidermidis*, and (C) *C. albicans*. In each plate, the center disc represents the negative control (chloroform). (D) Antibacterial activity of PHB (50% diluted with chloroform) against *S. aureus*, *S. epidermidis,* and *C. albicans* at various PHB concentrations. The error bar represents the mean ± standard deviation. (E) Optical densities of *S. aureus*, *S. epidermidis,* and *C. albicans* after treatment with different concentrations of PHB. *MIC concentrations of PHB. The MIC of PHB was calculated using the microdilution method. Broth was used as the negative control.

Disk diffusion was performed using concentrations of 0 (chloroform), 50, 100, 200, 400, 600, 800, and 1000 μg mL^−1^. The results showed (Fig. 8) decreased activity with low doses having no effect on the growth of *S. aureus, S. epidermidis* and *C. albicans*. An increase in concentration finally led to a 30 mm zone for S*. aureus* and a 28 mm zone for *S. epidermidis* at 1000 μg mL^−1^ concentrations, while a 20 mm zone was observed for *C. albicans* at the same concentration. This indicates that PHB has significant antimicrobial activity against staphylococcal species compared to *C. albicans*. Furthermore, a similar trend was observed with PHB when diluted to 50% (1 : 1 PHB:chloroform) to verify its antimicrobial activities (Fig. 8D). This also leads to the conclusion that PHB has a dose-dependent effect on all three tested strains. Additionally, protein and DNA leakage assays were performed using *E. coli* and *S. aureus* treated with PHB extracts. OD260 measurements revealed a 2.8-fold increase in extracellular DNA content after 4 hours of exposure, while treated samples showed a 3.2-fold increase in protein concentration in the supernatant. These results indicate a significant increase in extracellular protein and nucleic acid concentration compared to the untreated controls, confirming that membrane integrity was compromised. These results provide direct evidence of membrane disruption as a likely antimicrobial mechanism of PHB.

### 3.7 Minimum inhibitory concentration of PHB using the microdilution method

The MIC of PHB against each bacterium and fungus was calculated using the 96-well microdilution method (Fig. 8E). The wells were supplemented with PHB at 0, 200, 400, 600, 800 and 1000 μg mL^−1^. The PHB concentration at 0 μg mL^−1^ was the negative control. The results were calculated according to the CLSI protocols, which state that the concentration at which the OD value is less than or equal to 0.1 is chosen as the MIC value. The pattern observed in the cases of *S. aureus* and *S. epidermidis* indicates a dose-dependent effect. As the dose increases, the inhibition rate increases. In the case of *C. albicans*, the effect is again dose-dependent; however, the inhibition rate is comparatively reduced, which may be due to its structural components. The MIC value of PHB against *S. aureus* is 400 μg mL^−1^, *S. epidermidis* is 600 μg mL^−1^, and *C. albicans* is 1000 μg mL^−1^, as the OD values in this case are ≤0.1.

### 3.8 Time-kill assay

**Fig. 9:**
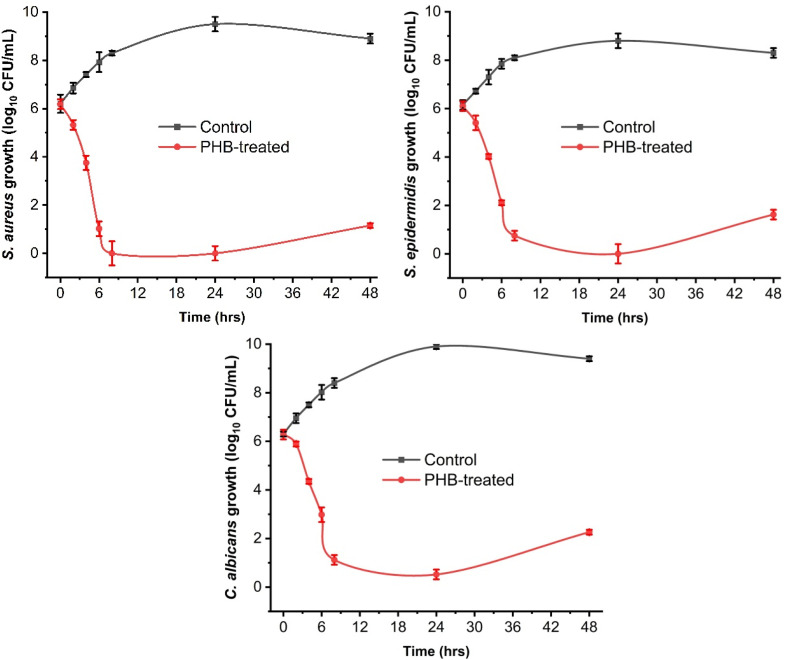
Time kill assay of *S. aureus, S. epidermidis* and *C. albicans* treated with PHB (MIC conc.). The data presented are the growth patterns using log10 CFU ml^−1^. The positive control used was broth and bacteria, and the negative control was broth alone.

The time-kill assay was performed using MIC concentrations (400 μg mL^−1^, 600 μg mL^−1^, and 1000 μg mL^−1^ for *S. aureus*, *S. epidermidis*, and *C. albicans*, respectively) on the selected strains (Fig. 9). The time-kill assay was studied as time-dependent growth kinetics. The samples were spread on NA plates after 2, 4, 6, 8, 24 and 48 h. The results are shown as log10 CFU mL^−1^. The time kill assay demonstrates the potent and broad-spectrum antimicrobial activity of PHB against all three tested pathogens. For *S. aureus*, PHB treatment causes a rapid bactericidal effect, reducing viability from 6.18log10 CFU mL^−1^ to complete eradication (0 CFU mL^−1^) within 8 hours. The plates were also analysed at 24 and 48 hours, and slight regrowth of the sample containing PHB was observed. The effect was similarly pronounced against *S. epidermidis*, leading to nearly complete elimination (0.75 log10 CFU mL^−1^) by 8 hours. The plates were also analysed at 24 and 48 hours, and slight regrowth of the sample containing PHB was observed but more than that of *S. aureus*. Although C. albicans showed greater inherent resistance compared to the staphylococcal species, PHB still achieved a substantial 5 log reduction from 6.28 to 1.12L log 10 CFU mL^−1^ over the same time. The overall comparison between the three strains implies that C. albicans showed a comparatively reduced effect on its growth.

### 3.9 Antibiofilm activity of the extracted PHB

**Fig. 10:**
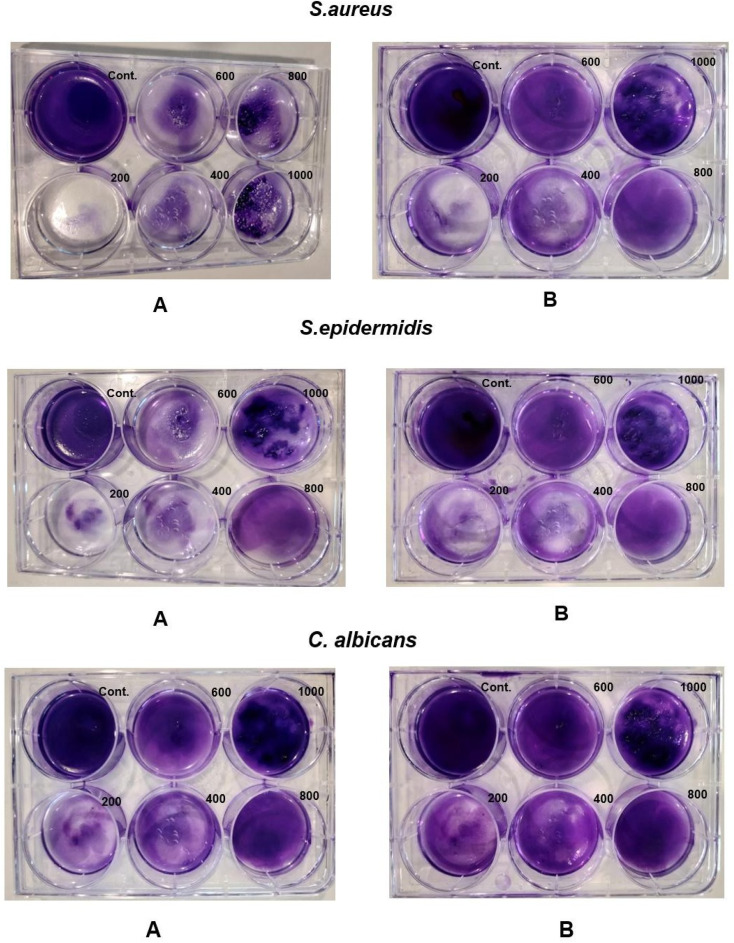
Antibiofilm activity of PHB against *S. aureus, S. epidermidis,* and *C. albicans* using CV assay. (A) represents co-incubation (inhibition of the biofilm); (B) shows eradication of the preformed biofilm. The positive control broth + bacteria without the drug was used in both figures (A and B).

The antibiofilm results were achieved by calculating the inhibition percentage. This was covered in two aspects: biofilm inhibition (co-incubation) and biofilm eradication (preformed biofilm). The method used was the crystal violet assay. In this case, the positive control was the bacterial culture without PHB. In the case of co-incubation, significant results (also shown in Table 2) were observed, with 60% of the biofilm being inhibited in the case of *S. aureus* and *S. epidermidis* at their MIC concentrations, 400 μg mL^−1^ and 600 μg mL^−1^, respectively. Almost 50% of the biofilm was inhibited in the case of *C. albicans* at an MIC value of 1000 μg mL^−1^, which indicates that *C. albicans* requires high doses. Fig. 10 shows a gradual decline in the colour of crystal violet, indicating significant activity against the tested strains (A), inhibition of biofilm (B), and eradication of biofilm.
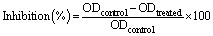

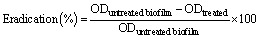


**Table 2:** Biofilm inhibition (co-incubation) by PHA against *S. aureus*, *S. epidermidis* and *C. albicans*

PHB concentration (μg mL^−1^)	% Inhibition			Key observations
	*S. aureus*	*S. epidermidis*	*C. albicans*	
0 (Control)	0%	0%	0%	Full biofilm formation
200	40%	30%	15%	Weak inhibition of fungi
400	60%	45%	20%	Near-MIC for *S. aureus*
600	75%	60%	30%	MIC for *S. epidermidis*
800	85%	70%	40%	Strong bacterial inhibition
1000	90%	80%	50%	MIC for *C. albicans*

Table 3 shows the results of the biofilm, where eradication was 60% in the case of *S. aureus* and *S. epidermidis* greater than MIC concentrations of 800 μg mL^−1^ and 1000 μg mL^−1^. Almost 35% of the biofilm was inhibited in the case of *C. albicans* at an MIC value of 1000 μg mL^−1^. This is because intact biofilms were already formed. The inhibition rate in the case of eradication is comparatively less in all three cases plausibly due to its maturation stage, EPS formation and strong interaction. *C. albicans* demonstrated a decrease in inhibition maybe due to its extensive hyphal nature.

**Table 3:** Biofilm eradication (preformed biofilm) by PHA against *S. aureus, S. epidermidis and C. albicans*

PHB concentration (μg mL^−1^)	% Eradication			Key observations
	*S. aureus*	*S. epidermidis*	*C. albicans*	
0 (control)	0%	0%	0%	Mature biofilm intact
400	30%	20%	10%	Minimal disruption
600	45%	35%	15%	Partial degradation
800	60%	50%	25%	Effective for bacteria
1000	70%	60%	35%	Best achievable with PHB alone
1000 + DNase	85%	75%	—	Synergy with enzymes

### 3.10 Assessment of biofilm inhibition and eradication using cell viability assay/XTT assay

**Fig. 11:**
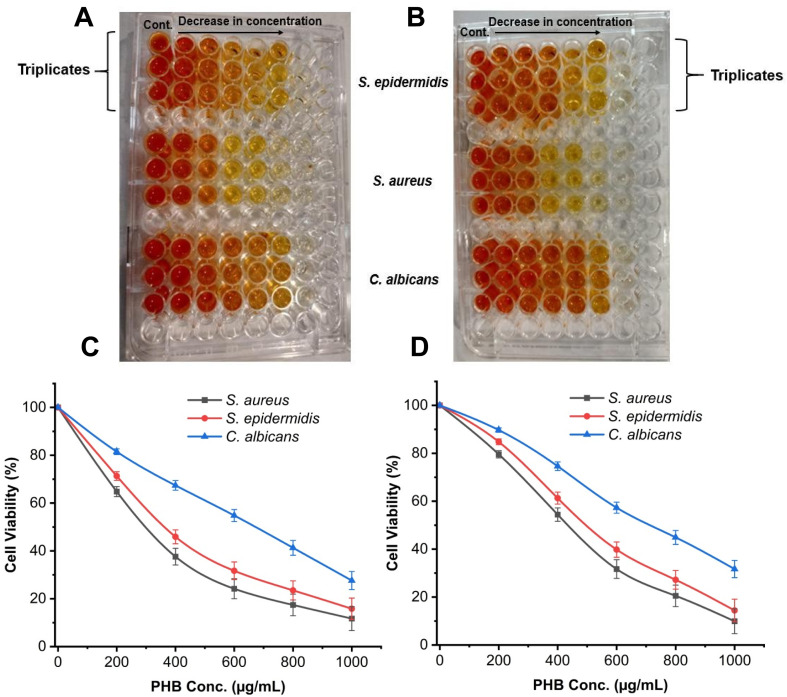
Antibiofilm activity of PHB against *S. epidermidis*, *S. aureus, and C*. *albicans* analysed using XTT assay. (A) Inhibition of the biofilm (co-incubation); (B) eradication of the biofilm (preformed). The experiment was run in triplicate. (Names indicated are in the following order: top to bottom). The positive control broth + bacteria without the drug was used in both figures (A and B). (C) Biofilm inhibition (co-incubation) by PHB and (D) biofilm eradication (preformed on pre-established biofilm) by PHB (right) against *S. aureus*, *S. epidermidis* and *C. albicans* using XTT assay. Data represent mean cell viability, and the error bar represents the standard deviation of the three biological replicates. The positive control broth + bacteria without the drug was used in both figures (C and D).

The viable cells in the biofilm were assessed using the XTT assay. A 96-well plate was used for this purpose, in which 6 concentrations were used. 0 μg mL^−1^ served as a positive control, leading to 100% biofilm formation in all the three cases. The results were divided into two aspects: co-incubation and preformed biofilm. The results showed a significant decrease in the viability of the planktonic cells. In the case of co-incubation, at MIC values of 400 μg mL^−1^ for *S. aureus*, 600 μg mL^−1^ for *S. epidermidis*, and 1000 μg mL^−1^ for *C. albicans*, cell viability was recorded as 37.6%, 31.7%, and 27.6%, respectively. The decrease in colour intensity can be observed in Fig. 11, part A. In the case of eradication of biofilm at MIC values, the viability results were 54.4%, 39.8%, and 31.7% for *S. aureus*, *S. epidermidis*, and *C. albicans*, respectively. This can be observed as a colour gradient in Fig. 11, part B. The results align with those of the crystal violet assay, showing significant activity of PHB in the case of co-incubation rather than pre-formed biofilms. Additionally, in both cases, the *anti*-biofilm activity against *Staphylococcus* species was significantly greater than that against *C. albicans*.
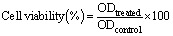


## 4. Discussion

The present study successfully optimized PHB production conditions and characterized polyhydroxyalkanoates. The optimization of production parameters and the evaluation of antimicrobial and antibiofilm properties provide significant insights into its potential applications in the healthcare sector. Our findings align with and expand upon the current understanding of microbial PHB production and its therapeutic potential. The strain provided in our study showed high PHB intensity (++++) when grown on MSM, followed by the Black Sudan B assay. This is similar to the results of,^33^ where *Bacillus axaraqunsis* BIPC01 was selected as a good PHB producer with (++++, intensity). An additional study selected *Bacillus* spp as robust PHB producers based on PHB accumulation using Black Sudan B.^51^

The orthogonal experimental design (OED) used in this study proved particularly effective for studying the effects of multiple variables and optimizing them simultaneously. In the present study, 7 factors, nitrogen source, carbon source, citric acid, NaCl stress, inoculum age, agitation speed, and incubation time, were optimized. A similar study was performed for PHB production by *Bacillus cereus* using this OED, focusing on 4 factors. Our results showed that *B. megaterium* was able to produce maximum PHB (46.7%, 4 g L^−1^) under the following conditions: ammonium sulphate (2 g L^−1^), glucose (20 g L^−1^), 10% NaCl, and 67 h incubation at 37 °C, with the inoculum age of the seed culture being 18 h at an agitation speed of 120 rpm. These results align with those of multiple studies. In a study, similar optimization of factors was performed to produce PHB from a moderately salt-tolerant strain of *B. megaterium*, where 2% glucose, 5% w/v NaCl, a temperature of 37 °C, and an incubation time of 54 hours produced 39% PHB.^52^ The productivity in the present study was high compared to that of,^53^ where *S. degradans*, a lignocellulosic degrader, was used for PHB production with glucose as the carbon source, producing 17.2% PHB. Additionally, one more study used ammonium sulphate as the nitrogen source, which led to a yield of 54.82% by *Pseudomonas* spp when 2% sucrose was also added. This study was in contrast to the present study, as 2 g L^−1^ nitrogen was sufficient to approximately yield these results.^54^

Citric acid supplementation appeared to enhance PHB production, which may be attributed to its potential role in increasing intracellular acetyl-CoA availability. As a key intermediate of the tricarboxylic acid (TCA) cycle, citric acid can be metabolized into acetyl-CoA, thereby supporting the biosynthetic pathway of PHB. Although direct quantification of acetyl-CoA flux was not performed, the observed improvement in polymer yield suggested a positive metabolic shift. Similar findings have been reported in earlier studies,^55^ where organic acid supplementation influenced central carbon metabolism and favored PHB accumulation. However, this proposed mechanism remains hypothetical in the current context, and further validation through metabolic profiling or isotopic labeling is beneficial for confirming the exact biochemical pathway. The results of this study helped us develop a plausible mechanism for the role of citric acid in increased PHB accumulation. The provided nitrogen-limited condition ultimately leads to a decline in AMP, which is required by the ICDH enzyme to convert citrate to α-ketoglutarate. Due to the decrease in nitrogen content, a reduction in ICDH activity results in more citrate accumulation, and, consequently, more acetyl Co-A. This finally enters the PHB synthesis pathways. This was also mentioned in 55, which showed PHB synthesis when glucose was used as a carbon source.

Additionally, when we supplemented citric acid, it caused a shift in metabolic flux from the TCA cycle towards biosynthetic pathways for PHB production, thereby increasing its productivity. Citric acid plays a central role in regulating PHB accumulation by acting as a vital metabolic hub that links carbon metabolism, energy production, and precursor availability. As a key intermediate in the TCA cycle, citric acid can be redirected towards PHB biosynthesis when metabolic conditions favour storage over growth (this study focuses on citric acid's role). Under nutrient-limited conditions, particularly nitrogen stress, the decline in AMP inhibits isocitrate dehydrogenase (ICDH), which results in citrate accumulation that is subsequently exported to the cytosol. Therefore, ATP citrate lyase (ACL) cleaves citrate into oxaloacetate and acetyl-CoA, with the latter serving as the direct substrate for PHB synthase. This metabolic flux is further amplified by citrate's ability to regulate glycolytic flux through inhibition of PFK (phosphofructokinase), effectively redirecting carbon away from catabolic pathways and towards PHB synthesis.

Furthermore, citrate helps maintain the cellular redox balance by connecting the TCA cycle with fatty acid metabolism, enabling flexible carbon sourcing from both sugars and lipids. The AMP-dependent regulation of citrate metabolism ensures that this process is closely linked to cellular energy status, promoting PHB production when energy levels are low. In practical terms, supplementing fermentation media with 2 g L^−1^ citric acid has been shown to significantly increase PHB yields, as demonstrated in experiments in which optimal citrate levels correlate with enhanced polymer accumulation. The multiple roles of citric acid make it a crucial “metabolic switch” that can be manipulated to optimise PHB production in bacterial systems under stress conditions. To our knowledge, the connection between citric acid and PHB in relation to metabolic pathways is reported for the first time.

PHB was extracted using the sodium hypochlorite method, followed by purification using the chloroform method coupled for pure yield. The method led to the extraction of a PHB yield of 4 g L^−1^. The methods used for PHB recovery were similar to those used by,^56^ but the solvent method used was non-halogenated, which is different from the one implemented in the present study. In another study, the extraction method used chloroform, which aligned with our study and showed an efficiency of 80% recovery, but degradation of the compound was also observed.^57^

In the present study, characterization was performed using two techniques: FTIR and NMR. The peaks were consistent with the results reported by other researchers on PHB characterization.^57^ FTIR analysis revealed characteristic PHB peaks at 1720 cm^−1^ (C=O stretch) and 2930 cm^−1^ (methylene groups), which are consistent with the spectra reported by.^54^^1^H-NMR signals at 1.26 ppm (–CH_3_) and 5.25 ppm (–CH) confirmed the poly-3-hydroxybutyrate (PHB) structure, matching reference data from.^57^ The ^13^C NMR spectra of PHB showed characteristic carbon signals at ∼179 ppm (carbonyl), ∼67 ppm (methine), ∼40 ppm (methylene), and ∼29 ppm (methyl), confirming the typical backbone structure of poly(3-hydroxybutyrate). These chemical shifts closely matched those reported for standard PHB, indicating high structural purity. The absence of unexpected peaks further supports the successful biosynthesis of PHB, with minimal impurities or side products. These analytical results validate the successful extraction and purification of PHB from *B. megaterium*, with spectral features comparable to the commercial PHB standards mentioned in 58 and 59.

The combined thermal and structural analyses provide comprehensive insight into the physicochemical properties of PHB synthesized from *B. megaterium*. Thermogravimetric analysis revealed that PHB exhibited higher thermal stability, with a maximum degradation temperature of 415 °C, which is significantly greater than that of the standard PHB. This suggests enhanced resistance to thermal breakdown likely due to differences in polymer chain length or structural integrity. Complementing this, DSC analysis showed similar melting points (∼167 °C) but notable variation in glass transition and cold crystallization temperatures. The higher Tg values observed in the microbial PHBs suggest restricted molecular mobility, which is possibly linked to lower chain regularity or amorphous regions. Crystallinity values calculated from melting enthalpies further supported these trends, with *B. megaterium*-derived PHB exhibiting a higher degree of crystallinity (44%). XRD analysis confirmed the semi-crystalline structure of PHB, with characteristic orthorhombic diffraction peaks. These findings highlight that *B. Megaterium* produces structurally comparable PHB, and differences in thermal behavior and crystallinity may influence their suitability for specific applications, such as biodegradable packaging or biomedical materials.

The extracted PHB demonstrated significant antimicrobial activity against skin pathogens, with zones of inhibition measuring 16 mm for *S. aureus*, 14 mm for *S. epidermidis*, and 10 mm for *C. albicans* at 1000 μg per disk. These results are comparable with the research conducted by,^60^ which achieved zones around 19.5 mm against *S. aureus* when the PHB film was fabricated with 3OHC15. These results compare favorably with previous studies on PHB antimicrobial properties. The MIC values, 400 μg mL^−1^ for *S. aureus*, 600 μg mL^−1^ for *S. epidermidis*, and 1000 μg mL^−1^ for *C. albicans*, suggest concentration-dependent activity, contrasting with the findings of,^61^ where MIC was 0.056 mg mL^−1^ against *P. aeruginosa* and *E. coli*, but it was because PHB alone was not used; rather, it was supplemented with the Ag/Cu nanocomposite. The time-kill assays showed complete eradication of *S. aureus* and *S. epidermidis* within 8 hours and of *C. albicans* within 10 hours. The activity was monitored for up to 24 hours, and no significant growth was observed. This supports the potential of PHB as an antimicrobial agent though the mechanism requires further investigation. These results, in contrast, vary from the results provided by^20^ against *E. coli* and *S. aureus*, which stated that significant growth inhibition (bactericidal effect) was observed during the 2–4 hours compared to 6–24 hours.

The antibiofilm activity demonstrated 60% inhibition of preformed biofilms at MIC concentrations, with enhanced effects at higher concentrations (70% eradication at 1000 μg mL^−1^). These results are comparable to those of,^62^ where 13% inhibition of biofilm formed by *Streptococcus pneumoniae* was observed; this work discussed similar biofilm disruption by microbial PHAs. The differential activity against bacterial *versus* fungal biofilms may relate to structural differences in extracellular matrices because fungus has a different structure, as reported in 63.

The significant *anti*-biofilm activity of PHB observed in this study aligns with emerging evidence on biopolymer-mediated microbial inhibition. Our findings demonstrating dose-dependent biofilm suppression (64.8% to 11.7% viability for MRSA at 200–1000 μg mL^−1^) corroborate previous reports of polyhydroxyalkanoates disrupting quorum sensing and extracellular polymeric substance production in Gram-positive bacteria (Patel *et al.*, 2015).^51^ The superior efficacy against staphylococcal species compared to *C. albicans* (27.6% *vs.* 31.7% viability at 1000 μg mL^−1^ in co-incubation and eradication assays, respectively) mirrors observations by,^64^ which reported that if mcl-PHA is used, its activity against *C. albicans* is significant at 500 μg mL^−1^. The results of the present study can be attributed to enhanced drug resistance mechanisms in fungal biofilms.^65^ The ability of PHB to achieve near-complete eradication of preformed MRSA biofilms (9.9% viability at 1000 μg mL^−1^) is particularly noteworthy, as it surpasses the performance of many conventional antibiotics. To the best of our knowledge, this study is the first to use the XTT assay to assess cell viability with scl-PHA. XTT has previously been performed for plant extracts.^66^ Additionally, the present study suggests that PHB may target fundamental biofilm stability factors potentially through physical disruption of the matrix, as mentioned in 67. In the present study, the residual *C. albicans* viability (31.7%) at maximum concentrations differed from that of,^64^ where PHB alone was not able to eradicate the biofilm produced by *C. albicans*. This indicates that for complete eradication, fungal biofilms may require combinatorial approaches, as mentioned in 68. The concept of the combinatorial approach is well explained in 69.

These results prove that the significant effect of microbial-derived PHAs represents a promising alternative for combating biofilm-associated infections, particularly for skin infections, both bacterial and fungal, where current therapies often fail. Future research should explore its use in hospital settings as a spray formulation to reduce the dissemination of nosocomial infections or healthcare-associated infections (HAIs), study structure–activity relationships of different PHB monomers and investigate indwelling device-related staphylococcal infections. Additionally, *in vivo* efficacy in biofilm infection models is needed to translate these findings into clinical applications.

## 5 Conclusion

This study demonstrated the production, optimization, and characterization of PHA, followed by its antimicrobial and antibiofilm potentials. In the initial phase of the study, PHB production was optimized by evaluating the effects of multiple nutritional and physical factors. Seven variables, including carbon and nitrogen sources, were systematically studied using an orthogonal experimental design (OED). The optimized media formulation significantly enhanced the yield of PHB by *B. megaterium*, making it suitable for downstream processing and characterization. PHB was then extracted from the bacterial biomass using a combined sodium hypochlorite digestion and chloroform extraction method. This technique resulted in the efficient recovery of the polymer with minimal degradation. The extracted polymer was then subjected to Fourier-Transform Infrared Spectroscopy (FTIR) and Nuclear Magnetic Resonance (NMR) analysis. The spectra obtained from both methods confirmed the identity of the polymer as polyhydroxybutyrate (PHB).

The antimicrobial potential of the extracted PHB was assessed against three clinically relevant pathogens: *Staphylococcus aureus*, *Staphylococcus epidermidis* and *Candida albicans*. Initial screening using the disk diffusion assay reveals significant zones of inhibition, suggesting notable antimicrobial activity. The minimum inhibitory concentration of the polymer was subsequently determined, followed by time kill assays, to evaluate the bactericidal kinetics. The polymer exhibited time-dependent killing. Further evaluation of PHB bioactivity was conducted through antibiofilm assays. The ability of the polymer to inhibit and eradicate biofilms was analysed using a crystal violet assay. In both preventive and curative setups, PHB significantly reduced biofilm biomass in all three test organisms, with more potent activity against the Gram positive, staphylococcal species. To assess the viability of cells within the biofilm, the XTT reduction assay was performed under two conditions: co-incubation and preformed biofilm. The results from the XTT assay supported the crystal violet assay data, confirming that PHB reduces biofilm formation and impairs the metabolic activity of biofilm-embedded cells.

## Author contributions

Manal and Faiqa Munir contributed equally to this work. They were responsible for conceptualization, data curation, formal analysis, methodology, and writing the original manuscript draft. Waseem Safdar and Saeed Ahmed provided conceptualization, critical input on data interpretation, visualization, formal analysis, methodology, manuscript writing, revising and manuscript proofreading. Muhammad Tariq Navid supervised the project, was involved in funding acquisition, assisted with study methodology, and reviewed the manuscript. Iftikhar Ahmed, Mahwish Ali, and Abu Bakr Shabbir provided the resources, assisted with the methodology, and reviewed the manuscript. All authors read and approved the final manuscript.

## Conflicts of interest

There are no conflicts to declare.

## Data Availability

All data required are present in the manuscript.
